# Evaluation of *in vitro* and *in vivo* Biological Activities of *Cheilanthes albomarginata* Clarke

**DOI:** 10.1186/1472-6882-14-342

**Published:** 2014-09-20

**Authors:** Ramakanta Lamichhane, Se-Gun Kim, Amrit Poudel, Dipak Sharma, Kyung-Hee Lee, Hyun-Ju Jung

**Affiliations:** College of Pharmacy, Department of Oriental Pharmacy, & BK 21plus Program & Department of Smart Life-Care Convergence, Wonkwang University, Iksan, Jeonbuk, Republic of Korea

**Keywords:** *Cheilanthes albomarginata*, Antioxidant, Phenolic content, Anti-inflammation, Anti-adipogenic

## Abstract

**Background:**

The *Cheilanthes albomarginata* Clarke (CA), a fern belonging to Pteridaceae family, is found mainly in India, Nepal, Pakistan and Bhutan at an altitude of 1300–2700 m. It grows mostly in the rock crevices on slopes. Juice from the rhizome of CA has been used to treat peptic ulcer. In this study, the biological activities (antioxidant, anti-inflammatory, anti-adipogenic and anti-obesity) of the extracts of CA were investigated. The total phenolic content of each extract was quantified. This is the first report regarding the study of biological activities on CA.

**Methods:**

In the current study, the crude methanol and fractionated extract of the aerial part of CA were investigated for the antioxidant tests which were namely DPPH assay, hydrogen peroxide scavenging assay and nitrite scavenging assay. Their phenolic contents were measured by the Folin-Ciocalteu’s method.

*In vitro* anti-inflammatory and anti-adipogenic assays were evaluated against the RAW 264.7 macrophage cells and 3 T3-L1 cells respectively. The crude methanol extract and phenolic fraction (combination of ethyl acetate and butanol fraction) were studied for the *in vivo* anti-obesity test using male Sprague Dawley rats.

**Results:**

The ethyl acetate fraction showed the strongest DPPH radical scavenging (82.54 ± 0.48%), hydrogen peroxide scavenging (3.41 ± 0.21 mg/ml) and nitrite scavenging activity (61.39%). The highest phenolic content was found in the ethyl acetate fraction followed by the butanol fraction. The ethyl acetate fraction showed the highest *in vitro* anti-inflammatory and anti-adipogenic activities. From the *in vivo* study on rats, the crude methanol extract and phenolic fraction showed plasma triglyceride lowering activity as well as reduction of weight of adipose tissue in high fat diet induced obese rats.

**Conclusion:**

The current study suggests that the ethyl acetate and butanol extracts of CA are potential source for antioxidant, anti-inflammatory and anti-adipogenic remedies. In addition to that the results of *in vivo* studies evidenced the possibility of CA as a source of anti-obesity drug remedies.

## Background

The importance of medicinal plants to the human livelihood is unexplainable. Medicinal plants are the fundamental necessities to human health care needs since the beginning of human civilization. Nepal is blessed with rich and diverse plant biodiversity. It is extremely rich in floral diversity in proportion to its size due to its wide altitude variation (60–8848 m) [1]. A total of 5,856 species of flowering plants, 28 species of gymnosperms, 853 species of bryophytes and 380 species of pteridophytes have been recorded from Nepal [2, 3].

Pteridophytes, which occupy the unique position between non-seed bearing and seed bearing plants make an important contribution to earth’s plant diversity. Pteridophytes are known to man for more than 2000 years for their medicinal values. Pteridophytes are used in Homeopathic, Ayurvedic and Unani medicines and provide insecticides, antibiotics, food and ornamentation [4]. It has been reported that *Cheilanthes farinosa* (Forsk) Kaulf, a fern which is used to treat skin disorders also possessed strong anti-inflammatory and anti-nociceptive properties [5]. Radhika NK et al. found the plant *C. farinosa* to produce considerable cytotoxic in hepatoma cell line, Hep 3B without inducing substantial damage to non-cancerous cell line RAW 264.7 [6]. Similarly different biological activities have been reported from the fern.

This study is an investigation of biological activities of the fern *Cheilanthes albomarginata* Clarke (CA). CA belonging to the family Pteridaceae, grows in rock crevices on slopes at an altitude of 1300 – 2700 m. It is found mainly in India, Nepal, Pakistan and Bhutan [7]. Rhizome of CA bears tufts of hair and pointed scales. Stipes can grow up to 25 cm long. The leaves are glabrous, reddish brown, shiny, and furnished particularly below when young.

It has lanceolate white margined scales. Fronds may be bipinnatisect to bipinnate, deltoid to deltoid lanceolate covered with white waxy powder. CA is a type of farinose fern as it contains white or yellow coating on the lower surface of the leaf [8]. Flavonoids like Apigenin 7-methyl ether (genkwanin), rhamnocitrin and kumatakenin have been isolated from CA [9].

Traditionally, juice and paste from the CA rhizome are used to treat peptic ulcer, stomach disorders and external cuts and wounds [10]. In a study done by Ghimire et al., the “tharu community” of Nepal was found to use the pounded juice of rhizome and the root from CA as a remedy for peptic ulcers [11]. The aqueous and ethanol extract of CA has antibacterial activity against *Agrobacterium tumefaciens*, *Escherichia coli*, *Salmonella arizonae* and *Staphylococcus aureus* [12].

Reactive oxygen species (ROS) such as hydroxyl (OH^.^) and peroxyl radical (ROO^.^) and the superoxide anion (O_2_^.^) are constantly produced as a result of metabolic reaction in living systems [13]. At low or moderate concentration, ROS exert beneficial effects on cellular responses and immune function but at high levels, free radicals and oxidants generates oxidative stress, a deleterious process that can damage cell structure, including lipids, proteins, and DNA [14]. Most living organisms possess efficient enzymatic and non-enzymatic defense systems against excess production of ROS. However, different external factors (smoke, diet, alcohol, some drugs), and aging decrease the capability of such protecting systems, resulting in disturbances of the redox equilibrium that is established in healthy conditions. Therefore, antioxidants that scavenge ROS may be of great value in preventing the onset and/or the progression of oxidative diseases [13].

Inflammation is the reactive state of hyperemia and exudation from blood vessels with consequent redness, heat, swelling and pain in which a tissue manifests a response to physical or chemical injury or bacterial invasion [15]. Several medicinal plant species are commonly used in traditional medicine as anti-inflammatory remedies. A number of anti-inflammatory constituents have been isolated and characterized structurally and pharmacologically from different medicinal plants [16].

Obesity, which is a strong risk factor for the development of chronic diseases such as type-II diabetes and cardiovascular disease, is characterized by an increase in the number and size of adipocytes differentiated from precursor cells, pre-adipocytes. Recent researches suggest that the accumulated fat in obesity also leads to increased ROS production resulting in systemic oxidative stress, and also contributing to obesity linked chronic diseases [12]. A high fat diet (HFD) increases the release of TNF-α from the gut, alters mucosal immunity, activates mast cells, increases vascular permeability, and disrupts the intestinal basement membranes. HFD promotes inflammation and obesity by interacting with and altering gut microbiota composition [17]. Inhibition of adipocytes differentiation is suggested to be an important strategy for prevention and/or treatment of obesity [18].

To the best of our knowledge, there has been no works related to antioxidant, anti-inflammatory, anti-adipogenic, and anti-obesity studies of CA. The promising antioxidant activity of the plant guided us to evaluate the anti-inflammatory and anti-adipogenic activity. The supportive *in vitro* results led us to investigate the *in vivo* study. So, the main objective of this study is to explore the biological activities of CA.

## Methods

### Sample collection

Aerial parts of CA were collected in Kaski district, Nepal, during June/July 2011 and identified by Dr. Radhe Shyam Kayastha, PhD., Tribhuvan University, Nepal. The voucher specimens (332) were deposited in the Pharmacognosy Laboratory of Pokhara University, Lekhnath Municipality-12, Kaski, Nepal.

### Reagents and chemicals

Solvents including methanol, ethyl acetate (EtOAc), n-butanol (BuOH), and chloroform (CHCl_3_) were purchased from SK chemicals (Seongnam, Korea) and were of analytical grade. 1, 1-diphenyl-2-picryl-hydrazyl (DPPH), Dimethyl sulfoxide (DMSO) were purchased from Junsei chemicals (Tokyo, Japan), Sulfanilic acid, N-(1-napthyl) ethylenediamine dihydrochloride, Folin Ciocalteu reagent, Gallic acid and ascorbic acid from Sigma Chemical Co. (St. Louis, MO), and Hydrogen peroxide from Daejung Chemicals (Daejung, Korea).

### Cell culture and bioassay reagents

RAW 264.7 cells and 3 T3-L1 cells were purchased from the American Type Culture Collection (ATCC, Rockville, MD). Dulbecco’s modification of Eagle’s medium (DMEM) was purchased from Gibco® by Life Technologies Co. (Carlsbad, CA) and Fetal bovine serum (FBS) from Hyclone (Logan, UT). Assay kits of total cholesterol, triglyceride, HDL-cholesterol, Aspartate aminotransferase (AST), Alanine aminotransferase (ALT), Glutamic pyruvate transaminase (GPT), Gamma-glutamyl transferase (γ-GTP), Blood urea nitrogen (BUN), and Creatinine were purchased from Asan Pharm Co., Ltd (Whasung, Korea). (3-(4,5-Dimethylthiazol-2-yl)-2,5-diphenyltetrazolium bromide (MTT), Dexamethasone (DEX), insulin, 1-methyl-3-isobutylxanthine (IBMX), Nicotinamide adenine dinucleotide phosphate (NADPH), Oil red O, Xanthine oxidase, and Hydrogen peroxide were purchased from Sigma Chemical Co. (St. Louis, MO).

### Extraction and fractionation

The aerial parts of the plant CA were collected and shade dried at room temperature for one week. After cutting them into smaller sizes, hot extraction was carried using methanol at 50°C in Wonkwang University, Korea. The extracts were filtered through Whatman no. 1 filter paper and concentrated using rotatory evaporator. The crude dried methanol extract was suspended in water and subjected to fractionation using chloroform, ethyl acetate and butanol to obtain chloroform, ethyl acetate, butanol, and water fractions.

### Total polyphenol content

Total polyphenolic compounds were determined with Folin-Ciocaltue reagent according to the standard method of Singleton and Rossi with some modification [19]. The content of total phenolic compounds in CA extract was determined as milligram of Gallic acid equivalent (GAE).

### DPPH assay

DPPH radical scavenging activity of CA was measured using the method proposed by Hazra et al., with slight modification [20]. The percentage inhibition of DPPH radical was calculated by comparing the results of the test with those of the control (not treated with extract) using the following formula.


where, C = Absorbance of the control and T = Absorbance of the test sample

The IC_50_ value was determined by interpolation form the non-linear regression of plot of percentage of inhibition against the concentration of extracts, which is defined as the amount of extract needed to scavenge 50% of DPPH radicals.

### Hydrogen peroxide scavenging assay

The ability of different extracts to scavenge the hydroxyl radicals (OH^**.**^) was measured according to the method of Muller [21].

### Nitrite scavenging assay

The nitrite scavenging activity of the extracts was evaluated by the method of Kato et al. [22]. NaNO_2_ solution was used for the production of nitrate radical.

### Cell culture and viability

The RAW 264.7 murine macrophages were maintained at sub-confluence in a 95% air and 5% CO_2_ humidified atmosphere at 37°C. DMEM medium supplemented with 10% fetal bovine serum (FBS) was used for routine sub-culturing and *in vitro* experiments.

3 T3-L1 pre-adipocytes maintained in DMEM with 10% bovine calf serum at 37°C in a humidified atmosphere of 5% CO_2_. After 2 days of 100% confluence (Day 0), adipocyte differentiation was induced by differentiation/induction medium (DMII) containing 0.5 mM IBMX, 1 μM Dexamethasone, and 10 μg/ml insulin in DMEM containing 10% FBS. Two days after the initiation of differentiation (Day 2), the culture medium was replaced with DMEM supplemented with only 10 μg/ml insulin and 10% FBS. After that the medium was replenished every 2 days (Day 4, Day 6, and Day 8) with 10% FBS in DMEM. To examine the effects of CA extracts on differentiation of pre-adipocytes to adipocytes, cells were differentiated with differentiation media containing various concentrations of the plant extract. Cell viability was determined colorimetrically using an MTT assay.

### Oil red O staining

Oil red O staining was used to monitor lipid accumulation in differentiated adipocytes. On day 8, cells were stained with Oil red O. The cells were fixed with 10% formalin for 30 min. After that the formalin was removed and washed with 60% isopropanol. Then the lipid droplets were stained for at least 30 min at room temperature in a freshly diluted Oil Red O solution [0.5% Oil Red O solution in 60:40 (v/v) isopropanol:water]. After Oil Red O stain, cells were photographed using a phase-contrast microscope (Olympus CK, Tokyo, Japan) in combination of digital camera at 100 × magnifications. Finally, the dye retained in the 3 T3-L1 cells was eluted with isopropanol and quantified by measuring the absorbance at 510 nm.

### *In vitro*anti-inflammatory activity

RAW 264.7 cells were plated in a 24 well plate at a density of 10^6^ cells/mL, 500 μL in each well. Then after 24 hr incubation, the medium was changed and the samples (extracts of CA) were added. After 1 hr. of sample treatment LPS (final concentration: 1 μg/ml) was added to both extracts treated as well as untreated wells. Amount of nitrite produced were measured using Griess reagent (1% sulfanilamide and 0.1% napthylethylenediamine dihhydorchloride in 2.5% phosphoric acid). 100 μL of cell culture medium was mixed with 100 μL of Griess reagent. Subsequently, the mixture was incubated at room temperature for 10 min and the absorbance at 540 nm was measured in a microplate reader. Fresh culture media was used as a blank.

### *In vivo*assay

#### Preparation of samples

Crude methanol extract and phenolic fraction (mixture of butanol and ethyl acetate fraction in equal ratio of weight) were taken to prepare samples for the *in vivo* assay. The *in vitro* study evidenced the comparable activity for the butanol and ethyl acetate fraction. In addition, the TLC patterns for both fractions were similar. So we tried to see the *in vivo* effect of the combination of the two higher phenolic compound containing extracts. Required amount of extracts were weighed and suspended in PBS and homogenized using a homogenizer. The dose fed orally to each rat was 200 mg/kg per day.

#### Animals and experiment design

Four-week-old, male SD (Sprague Dawley) rats were purchased from Damool Science (Daejeon, Korea). The rats were maintained in accordance with the guidelines for the care and use of laboratory animals of Wonkwang University. All experiments complied with ethical standards and were approved by the Animal Ethics Committee at Wonkwang University (Iksan, Korea). They were housed under a 12-h light/12-h dark cycle in a temperature of 25 ± 2°C and humidity of 55 ± 5%. They were also allowed food and water ad libitum. After adaptation to the lighting conditions for a week, the rats were divided into four groups (each group 8 rats) as follows:

1. Normal diet (ND) or Normal group
2. High fat diet (HFD) or Control group
3. HFD-M group- high fat diet supplemented with MeOH extract
4. HFD-P group- high fat diet supplemented with phenolic fraction.

Body weights were recorded weekly. The amount of food intake was measured in every three days.

### Biochemical analysis on blood and evaluation of organ weight

After 8 weeks, all rats were fasted for 12 hr. prior to sacrifice. After the fasting, each rat was deeply anesthetized by an overdose of carbon dioxide and the blood was drawn from the posterior vana cava. The serum was separated by centrifuge and stored at −80°C until analysis. The fat (epididymal and retroperitoneal), liver, spleen and kidney were removed. Their weights were taken immediately and stored at - 70°C.

Total cholesterol (TC) and triglycerides (TG) content were determined with assay kit (AM 202-K, AM 157S-K Asan Pharm Co., Ltd, Whasung, Korea) according to the protocol obtained from manufacturer. High density lipoprotein cholesterol (HDL-C) content was analyzed with assay kit (AM 203-K, Asan Pharm Co., Ltd, Whasung, Korea). Liver function test was determined with commercial assay kits of Catalase (CAT), GPx (glutathione peroxidase) (Asan Pharm Co., Ltd, Whasung, Korea). Superoxide dismutase (SOD) activity was assessed according to the modified method of Oyanagui [23].

### Protein determination and statistical analysis

Protein quantification was measured by Lowry’s method with bovine serum albumin (BSA) as a standard [24]. All data are presented as mean ± SD of triplicate experiments. Statistical analysis was carried out by SPSS statistics 19 software (IBM Co., Armonk, NY) measured by one-way analysis of variance (ANOVA) using Duncan’s multiple range test. P values considered significance at the level of less than 0.05%.

## Results and discussion

### Total polyphenol content

Flavonoids like kumatakenin, kaempferol, rhamnocitrin, etc. have been isolated from CA. There may be many other flavonoids present in CA. The total constituent of polyphenol compounds in CA was observed by polyphenol content assay. The results of the polyphenol content assay are exhibited in Table 1. The total phenolic content in the plant extracts is expressed in terms of Gallic acid equivalent (GAE). The standard curve equation, y = 0.0073x + 0.0168, (R^2^ = 0.99) was used for the calculations. Table 1 summarizes that ethyl acetate fraction has the highest total phenolic content (216.08 ± 1.3 GAE mg/g dry extract), followed by butanol fraction, methanol crude extract, chloroform fraction and water fraction. Phenolic compounds have been known to possess high antioxidant properties due to their free radical scavenging properties [25]. The calculation of phenolic content helps to predict the antioxidant potential of the extracts. It has been reported that extract containing large amount of polyphenol content possesses a greater antioxidant activity [26]. The polyphenol content of methanol extract of CA was found two times greater than that of other ferns like *Cyathea latebrosa, Cibotium barometez, Drynaria quercifolia, Blechum orientale, Dicranopreris linearis, Diaplazium esculentum, Lygodium circinnatum, Nephrolepsis biserrata, and Pyrossia munulariofolia* [27]. So, CA can be considered as a higher polyphenolic compounds containing fern.

**Table 1:** **Total phenolic contents of crude methanol extract and other fractions of CA**

Fractions	Total phenolic content (GAE)
**MeOH**	51.2 ± 0.23
**CHCl**_**3**_	37.42 ± 0.25
**EtOAc**	216.08 ± 1.30
**BuOH**	93.15 ± 0.58
**Water**	24.78 ± 0.10

Each value is average of three analysis ± standard deviation.

### Determination of DPPH radical scavenging activity

DPPH radical assay is one of the most extensively used methods to evaluate antioxidant activity of plant extracts, foods and single compounds. This assay is based on the measurement of the reducing ability of antioxidants toward DPPH radical, and the decrease of its absorbance [28]. DPPH radical is stable and commercially available organic nitrogen radical, which reacts with hydrogen/electron donor compounds and has a maximum UV–Vis absorption within the range of 515–520 nm. Upon reduction, the radical solution becomes discolored. Table 2 gives the result from the DPPH radical assay in IC_50_. In our study, the highest scavenging effect was observed in the ethyl acetate fraction with an IC_50_ of 16.33 ± 0.48 μg/ml. This was followed by crude methanol extract, butanol, water and chloroform fractions. The ascorbic acid being a single compound showed a greater antioxidant activity. The highest antioxidant activity of ethyl acetate fraction is due to the presence of greatest amount of polyphenolic compounds in this fraction compared to others.

**Table 2:** **DPPH radical assay of crude methanol extract and other fractions of CA**

Sample	IC _50_(μg/ml)
**MeOH**	19.55 ± 2.83^bc^
**CHCl**_**3**_	38.0 ± 6.03^d^
**EtOAc**	16.33 ± 0.48^b^
**BuOH**	24.66 ± 1.35^bc^
**Water**	27.80 ± 3.52^c^
**Ascorbic acid**	5.36 ± 0.27^a^

Each value is average of three analysis ± standard deviation. Values with different letters are significantly different (p < 0.05) based on one-way ANOVA post-hoc Ducan Multiple Range tests.

### Hydrogen peroxide scavenging activity

Hydrogen peroxide is an intermediate during endogenous oxidative metabolism and mediates radical oxygen formation such as ^•^HO, which may be used to predict the scavenging capability of antioxidants in biological systems [29].

Although hydrogen peroxide itself is not very reactive, it can sometimes cause cytotoxicity by giving rise to hydroxyl radicals in the cell. Thus, removing H_2_O_2_ is very important throughout food systems [30]. The extracts of CA were capable of scavenging hydrogen peroxide in a concentration dependent manner. IC_50_ for scavenging of H_2_O_2_ was 3.41 ± 0.21 for ethyl acetate fraction which was the highest among the fractions. The scavenging of other fractions is given in Table 3. The IC_50_ value for standard butyl hydroxyanisole (BHA) was 1.32 ± 0.07.

**Table 3:** **Hydrogen peroxide scavenging assay of the crude methanol extract and other fractions of CA**

Sample	IC _50_(μg/ml)
**MeOH**	21.65 ± 1.73^b^
**CHCl**_**3**_	46.56 ± 4.81^c^
**EtOAc**	3.41 ± 0.21^a^
**BuOH**	6.73 ± 0.20^a^
**Water**	28.38 ± 2.20^b^
**BHA**	1.32 ± 0.07^a^

Each value is average of three analysis ± standard deviation. Values with different letters are significantly different (p < 0.05) based on one-way ANOVA post-hoc Ducan Multiple Range tests.

### Nitric oxide (NO) scavenging assay

NO is a potent pleiotropic inhibitor of physiological process, such as smooth muscle relaxation, neural signaling, inhibition of platelet aggregation and regulation of cell mediated toxicity. It is a diffusible free radical that plays many roles as an effector molecule in diverse biological systems including neuronal messenger, vasodilation and antimicrobial and antitumor activities [31]. However, in pathological situations NO is also known to injure cells and tissues at relatively high concentrations [32].

**Figure 1:**
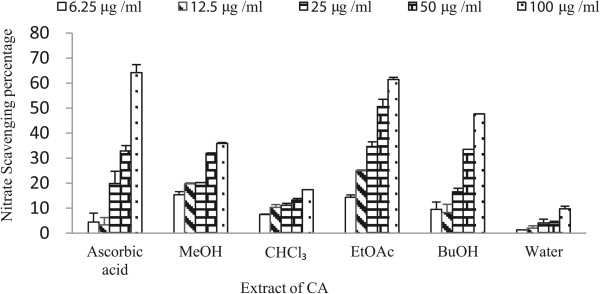
**NO scavenging activity of crude methanol extract and fractions of CA.** Data are expressed as mean ± S.D.

The results of NO scavenging assay are given in Figure 1 as nitrate scavenging percentage. Among the different extracts ethyl acetate extract showed the highest scavenging activity 61.39% at 100 μg/ml, whereas ascorbic acid exhibited 64.15% at the same concentration. This similar activity of a fraction and single compound indicates that the fraction contains potential antioxidant compounds similar to ascorbic acid. The results also showed there was an increase in activity with the increase in concentration of extract.

### Effects of CA on cell viability

**Figure 2:**
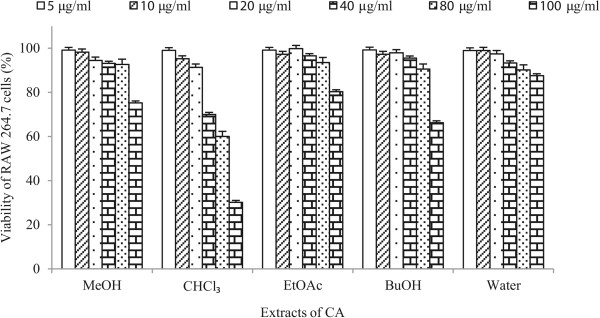
**Cell viability assay of the crude methanol extract and other fractions of CA on RAW 264.7 cells using five concentrations.** Data are expressed as mean ± S. D.

**Figure 3:**
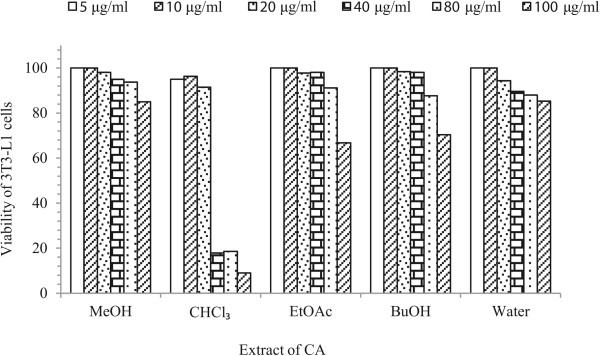
**Cell viability assay of the crude methanol extract and other fractions of CA on 3 T3-L1 preadipocyte cells.** Data are expressed as mean ± S. D.

In this study, methanol crude extract, ethyl acetate fraction, butanol fraction and water fraction up to a concentration of 80 μg/ml did not decrease the viability of RAW 264.7, and 3 T3 L1 cells (Figures 2 and 3). For the chloroform fraction, 20 μg/ml was found to be slightly toxic concentration for both types of cells. Therefore, concentrations up to 15 μg/ml for chloroform extract and 80 μg/ml for the remaining fraction were chosen for subsequent experiments.

### Inhibition of NO production in RAW 264.7 cells

**Figure 4:**
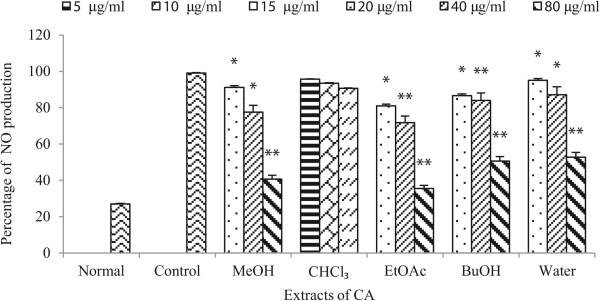
**Effect of CA extracts on NO production in LPS-stimulated RAW 264.7 cells.** RAW 264.7 cells were treated with LPS, with or without different concentration of extracts. The culture supernatant was analyzed for nitrite production. NO production was measured after 20 hrs of sample treatment by the Griess reaction assay. Normal group was treated with media only. Control group was treated with the lipopolysaccharide (LPS, 1 μg/mL) alone. Data are expressed as mean ± S. D., where ^**^p < 0.001,^*^p < 0.05 compared with control.

To determine whether CA extracts have anti-inflammatory activity, the effect of each extract on the production of NO was evaluated. The Figure 4 shows the percentage of production of NO in sample treated and non-treated LPS-stimulated RAW 264.7 cells. There was significant highest inhibition of NO production in the ethyl acetate fraction followed by the methanol crude extract, butanol fraction, and water fraction. Chloroform extract did not show significant inhibition. The activity at 80 μg/ml was effective while there was no significance inhibition at 40 μg/ml and 20 μg/ml. The production of NO decreased on concentration dependent manner. From this we conclude that CA has also potential anti-inflammatory activity. NO is believed to be a major pro-inflammatory mediator concerned with pathogenic infections by bacteria and viruses.

### Effect of CA extracts on adipocyte differentiation

**Figure 5:**
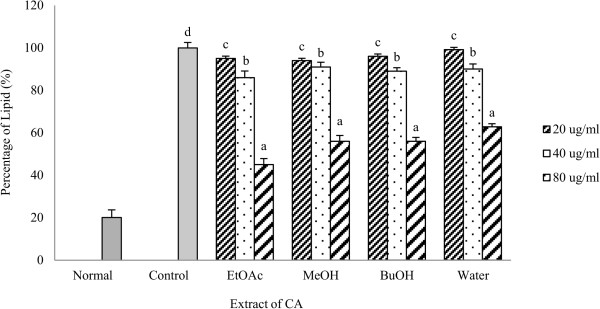
**Effect of CA extracts on adipogenesis of 3 T3-L1 cells.** The cells were treated with DMII alone (control) or with DMII and CA extracts. Normal group were cultured with normal media. The 3 T3-L1 cells were fully differentiated by 8 days, and the accumulation of lipids was measured by Oil red O staining. Each value is average of three analysis ± standard deviation. Mean with different letters indicate significant differences at p < 0.05 compared to the control according to one-way ANOVA post-hoc Ducan Multiple Range tests.

Figure 5 shows the results of production of lipids in 3 T3-L1 cells treated with DMII alone or with DMII and CA extracts. The plant has also the potential to inhibit lipogenesis which can be figured out form the results in Figure 5. The cells treated with the DMII mixture plus 80 μg/ml of each extract separately resulted in 43% (MeOH), 52% (EtOAc), 37% (BuOH) and 36% (water) reduction of the lipid droplets respectively. The activity decreased with the concentration. The results for chloroform extract are not shown as it showed no significant activity. Very few or no research has been done related to anti-adipogenesis effect of ferns. So this study has been a representative study giving an idea that fern has also potential to show anti adipogenic activity.

**Figure 6:**
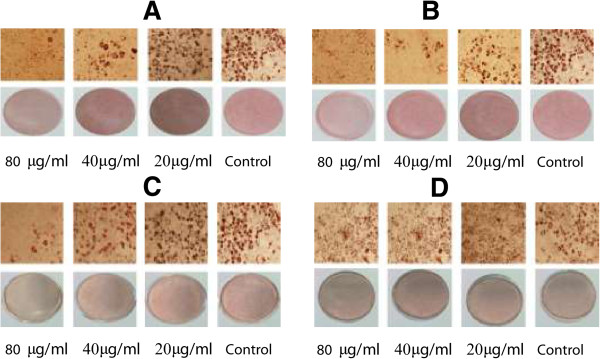
**Oil red O stained 3 T3-L1 adipocytes treated with DMII and extracts {MeOH extract (A), EtOAc extract (B) BuOH extract (C) and water extract (D)} or DMII alone (control).** The concentrations of samples were 80, 40, and 20 μg/ml. The lipid produced after differentiation till Day 8 was stained with Oil red O staining agent and pictures were taken using microscope. The red circular bands are the lipid produced during differentiation.

Figure 6 consists of photos of the oil red O stained adipocytes taken by Olympus microscope (Tokyo, Japan). Figure 6A, 6B, 6C, and 6D show the effect of EtOAc, MeOH, BuOH and H_2_O extracts on the accumulation of lipid in 3 T3-L1 cells respectively at different concentration. The control group which was treated with the differentiation media only (DMII) is also shown together. The red spots are the regions of lipid accumulation which is visible when stained by oil red O. We can see suppression of adipogenesis (decrease in staining) with the increase in concentration of the extract. The EtOAc fraction (A) showed a greater suppression compared to other fractions.

### *In vivo*assay (Animal experiment)

#### Body weight, food intake and food efficiency ratio

The effect of extracts on the body weight gain, food intake and food efficiency were examined in Table 4. The final body weight of the HFD group was significantly higher than that of the ND group. However, in the group fed with crude methanol extract (HFD-M) and phenolic fraction (HFD-P) the final body weight was decreased by 19.04% and 14.81% respectively. Since there was not so difference in the food intake among the control and sample groups, the results indicated that the treatment of extract did not affect the food intake. The reduction of body weight gain was not due to food intake pattern but due to the treatment of extracts. These results showed that the extracts play role in burning fat in the body and prevented the weight gain.

**Table 4:** **The effect of extracts on the body weight, weight gain, food intake and feed efficiency body weight gain = final body weight - initial body weight**

Group	Dose (mg/kg)	Food intake (g/day)	Initial body weight (g)	Final body weight (g)	Body weight gain(g)	Feed efficiency ratio (FER)
**Normal**		16.21 ± 3.62^a^	129.73 ± 3.22^a^	342.32 ± 8.51^a^	212.72 ± 6.42^b^	0.17 ± 3.62
**Control**		15.53 ± 1.21^b^	131.31 ± 4.12^ab^	378.72 ± 10.03^b^	248.31 ± 12.71^b^	0.28 ± 1.23
**HFD-M**	200	14.95 ± 1.82^b^	126.15 ± 6.74^b^	306.37 ± 31.56^bc^	180.25 ± 24.81^bc^	0.21 ± 1.84
**HFD-P**	200	15.64 ± 1.61^b^	119.25 ± 2.31^c^	322.62 ± 38.45^c^	203.37 ± 36.14^c^	0.23 ± 1.61

FER (Food Efficiency Ratio) = body weight gain (g/day)/food intake (g/day). Values were express as the mean ± SD. The effects of samples were compared by one-way analysis of variance (ANOVA) using Duncan’s multiple range test. Values with different letters of the column are significantly different (p < 0.05) based on one-way ANOVA post-hoc Ducan Multiple Range tests.

### Assessment of potential toxicological effects

To evaluate potential toxic effect of extracts, serum toxicological markers, which indicate liver and kidney injury, were measured at the end of the experimental period. The levels of GPT, AST, ALT, blood nitrogen urea and creatinine were not significantly changed in extract treated rats compared to HFD fed rats. Additionally, the extract treated rats did not induce significant changes in the weight of liver and spleen (data not shown). This indicates that oral administration of 200 mg/kg/day of the extracts for 8 weeks induced no detectable adverse toxic effects in rats.

### Weight of adipose tissue and serum lipids

**Figure 7:**
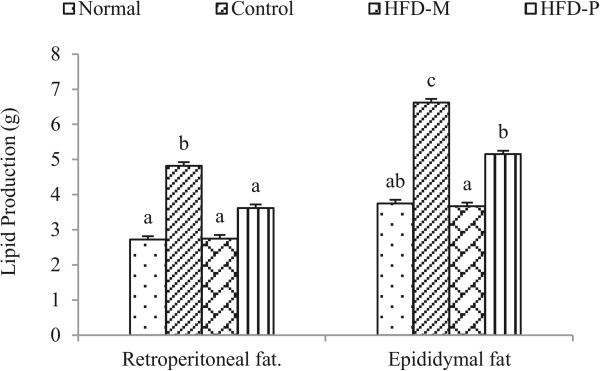
**Effect of CA extracts on change in Adipose tissue weight after 8 weeks.** The production of adipose tissues increased in control. In HFD-M and HFD-P group the fraction reduced the production of adipose tissue. Data were express as the mean ± SD. Mean with same letters indicate no significant differences at p < 0.05 according to one-way ANOVA post-hoc Ducan Multiple Range tests.

To investigate whether the extracts decrease adiposity, rats were sacrificed and adipose tissues were removed and weighed. The weight of adipose tissues: retroperitoneal and epididymal fat were increased in HFD group compared to the ND group. In case of HFD-M and HFD-P group the weight of adipose tissues were decreased (Figure 7).

**Figure 8:**
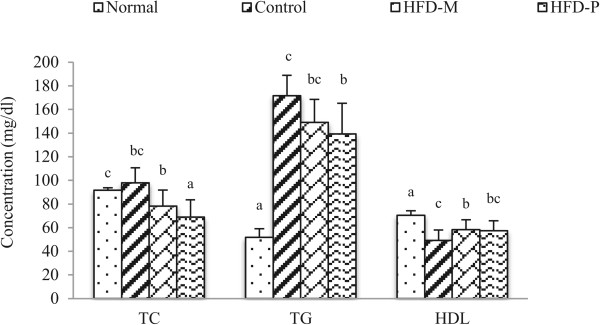
**Effects of extract on Serum lipids of rats fed with HFD.** Values were express as the mean ± SD. Mean with same letters indicate no significant differences at p < 0.05 according to one-way ANOVA post-hoc Ducan Multiple Range tests.

The Serum lipid profile is one important parameter for the analysis of obesity. Serum lipid profiles of normal and HFD groups are summarized in Figure 8. The increase in Total cholesterol (TC) and Triglyceride (TG) in HFD group compared to normal group indicated an induction of obesity. The TG in the HFD group (166.7 ± 17.3 mg/dL) was increased significantly compared to the normal group (53.49 ± 7.41 mg/dL). The HFD-M and HFD-P group showed reduction of the HFD induced increased triglyceride and total cholesterol levels. The HFD-P group showed larger reduction of plasma lipid compared to the HFD-M (Figure 8). There is increase in the level of HDL in HFD-M and HFD-P group compared to the HFD group. The serum lipid analysis showed the lipid lowering potential of CA extracts. Among the two extracts, the phenolic fraction showed higher lipid lowering potential than the methanol extract.

### Effect on activity of hepatic enzymes

**Figure 9:**
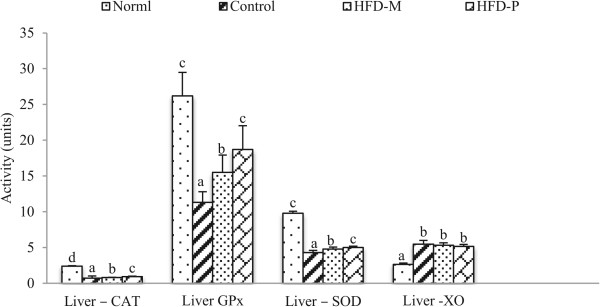
**Effect of extracts on activity of hepatic enzymes: Catalase (CAT), glutathione peroxidase (GPx) and superoxide dismutase (SOD) and Xanthine oxidase (XO).** The administration of fractions (HFD-M and HFD-P group) for 8 weeks increased the level of antioxidant enzymes CAT, GPx and SOD and decreased the level of free radical generating enzyme XO. Values were express as the mean ± SD. Mean with same letters indicate no significant differences at p < 0.05 according to one-way ANOVA post-hoc Ducan Multiple Range tests.

The important antioxidant enzymes necessary in all oxygen metabolizing cells are catalase (CAT), glutathione peroxidase (GPx) and superoxide dismutase (SOD). The SOD converts superoxide radical into hydrogen peroxide and molecular oxygen. Then catalase and GPx convert hydrogen peroxide into water. On the other hand xanthine oxidase (XO) is a free radical generating enzyme [33]. The level of antioxidant enzymes (Catalase, GPx and SOD) are lower and that of XO is higher in HFD group compared to normal group. This indicates that the obesity resulted in the decrease of activity in the antioxidant enzymes and increased in the free radical generating enzyme. When the extracts were administered (HFD-M and HFD-P group), the level of antioxidant enzymes increased. The increase in GPx activity was the highest compared to other antioxidant enzymes. There was no significant reduction of the free radical generating enzyme XO (Figure 9). However, the significant increment in the activity of antioxidant enzymes indicates that the extracts have some role for *in vivo* antioxidant activity. The results (Figure 9) showed the phenolic extract being more potent than the methanol extract for the *in vivo* antioxidant activity. The higher polyphenol content of phenolic fraction may be the reason for its higher *in vivo* antioxidant activity.

## Conclusion

The plant CA has been traditionally used as remedies for peptic ulcer, cuts, wounds and stomach problems. This present study was designed to examine antioxidant, anti-inflammatory, and *in vivo* anti-obesity activity.

The polyphenol content assay of CA extracts was followed by the antioxidant activity test. The EtOAc fraction, which was found to contain the highest polyphenol content, also showed the highest antioxidant, anti-inflammatory and anti-adipogenic activity as well. The *in vitro* activity of BuOH fraction was comparable to EtOAc fraction. The good *in vitro* antioxidant, anti-inflammatory and anti-adipogenic activity of CA guided us to evaluate the *in vivo* anti-obesity activity, since oxidative stress and inflammation are the important factors for inducing and promoting obesity [12, 17]. As there was comparable activity for the EtOAc and BuOH fractions, we prepared a phenolic extract sample (mixing EtOAc and BuOH fraction in equal ratio by weight), for the *in vivo* study.

It has been well known that the size of adipose tissue increases during obesity due to the accumulation of fats in the adipose tissue [34]. Our *in vitro* experiment showed the decrease in accumulation of fat droplets in 3 T3 L1 adipocytes when the cells were treated with CA extracts. Further the *in vivo* study revealed reduction in body weight, adipose tissue mass, TG and TC when the extracts of CA were supplemented with HFD. This indicates the anti-obesity activity of CA extract.

The literature survey showed very few number of research works about the anti-obesity activity on fern. So this study has played an important role to get an idea about anti-obesity activity of fern.

From the overall results we can conclude that the plant CA has antioxidant, anti-inflammatory and anti-obesity activity. So, further investigation and research work can play an important role to develop this plant as a remedy for the treatment of diseases associated with oxidative stress, inflammation and obesity.

## References

[1] Kindlemann P (1998). Himalayan Biodiversity in the Changing World.

[2] Iwatsuki K (1988). An Enumeration of the Pteridophyte of Nepal.

[3] Pradhan N, Joshi SD (2008). A diversity account of BRYACEAE (Bryophyte: MUSCI) of Nepal. J Nat Hist Mus.

[4] Hynniewta SR, Kumar Y (2008). Herbal remedies among the *Khasi* traditional healers and village folks in Meghalaya. Indian J Tradit Knowl.

[5] Yonathan M, Asres K, Assefa A, Bucar F (2006). *In vivo* anti-inflammatory and anti-nociceptive activities of *Cheilanthes farinosa*. J Ethnopharmacol.

[6] Radhika NK, Sreejith PS, Asha VV (2010). Cytotoxic and apoptotic activity of *Cheilanthes farinosa* (Forsk.) Kaulf. against human hepatoma, Hep3B cells. J Ethnopharmacol.

[7] Wu Z, Peter RH, Hong D (2013). Flora of China.

[8] Wollenweber E, Schneider H (2000). Lipophilic exudates of Pteridaceae - chemistry and chemotaxonomy. Biochem Syst Ecol.

[9] Eckhard W (1976). Flavonoid exudations in Farionse Ferns. Phytochemistry.

[10] Manandhar NP (2002). Plants and People of Nepal.

[11] Ghimire K, Bastakoti RR (2009). Ethnomedicinal knowledge and healthcare practice of Nawalparasi district in central Nepal. For Ecol Manag.

[12] Parihar P, Parihar L, Bohra A (2010). *In vitro* antibacterial activity of fronds (leaves) of some important pteridophytes. J Microbiol Antimicrob.

[13] Wang H, Nair MG, Strasburg GM, Chang YC, Booren AM, Gray JI, Deitt DL (1999). Antioxidant and anti-inflammatory activities of anthocyanins and theri aglycon, cyanidin, from tart cherries. J Nat Prod.

[14] Lee OH, Kwon YI, Apostolidis E, Shetty K, Kim YC (2010). Rhodiola-induced inhibition of adipogenesis involves antioxidation enzyme response associated with penstose phosphate pathway. Phytother Res.

[15] Ebrahimzadeh MA, Nabavi SM, Navavi SF, Bahramian F, Bekhrandia AR (2010). 2010. Antioxidant and free radical scavenging activity of *H. officinalis* L*.* Var. Angustifolius*, V. odorata, B. Hyracana and C. speciosum*. Pak J Pharm Sci.

[16] Pietta P, Simonetti P, Mauri P (1998). Antioxidant activity of selected medicinal plants. J Agric Food Chem.

[17] Lee C (2013). The effect of high-fat diet-induced pathophysiological changes in the gut on obesity: what should be the ideal treatment?. Clin Transl Gastroenterol.

[18] Khan RA, Khan MT, Sahreen S, Ahmed M (2012). Evaluation of phenolic contents and antioxidant activity of various solvent extracts of *Sonchus asper* (L.) Hill. Chem Cen J.

[19] Singleton VL, Rossi JA (1965). Clolrimetry of total phenolics with phosphomolybdic-phosphotungstic and reagents. Am J Enol Vitic.

[20] Hazra B, Sarkar R, Biswas S, Mandal N (2010). Comparative study of the antioxidant and reactive oxygen species scavenging properties in the extract of the fruits of *Terminalia chebula*, *Terminalia Belerica* and *Embilica officinalis*. BMC Complement Alter Med.

[21] Muller H (1985). Detection of hydrogen peroxide produces by microorganisms on an ABTS peroxidase medium. Zentralbl Bakteriol Mikrobiol Hyg A.

[22] Kato H, Lee IE, Chuyen NV, Kim SB, Hayase F (1987). Inhibition of nitrosamine formation by nondialzable melanidines. Agric Biol Chem.

[23] Oyanagui Y (1984). Reevaluation of assay methods and establishmednt of kit for superoxide dismutase activity. Anal Biochem.

[24] Lowry O, Rosebrough N, Farr A, Randall R (1951). Protein measurement with folin phenol reagent. J Biol Chem.

[25] Evans CR, Miller N, Pagana G (1997). Antioxidant properties of Phenolic compounds. Trends Plant Sci.

[26] Proestos C, Boziaris IS, Nychas GJE, Komaitis M (2006). Analysis of flavonoids and phenolic acids in Greek aromatic plants: Investigation of their antioxidant capacity and antimicrobial activity. Food Chem.

[27] Lai HY, Lim YY (2011). Evaluation of antioxidant activities of the methanolic extracts of selected ferns in Malaysia. Int J Environ Sci Dev.

[28] Prior RL, Wu X, Schaich K (2005). Standardized methods for the determination of antioxidant capacity and phenolics in foods and dietary supplements. J Agric Food Chem.

[29] Juntachote T, Gerghofer E (2005). Antioxidative properties and stability of ethanolic extracts of Holy basil and Galangal. Food Chem.

[30] Nabavi SM, Ebrahimzadeh MA, Nabavi SF, Hamidinia A, Bekhradnia AR (2008). Determination of antioxidant activity, phenol and flavonoids content of *Parrotia persica Mey*. Pharmacologyonline.

[31] Hagerman AE, Riedl KM, Jones GA, Sovik KN, Ritchard NT, Hartzfeld PW (1998). High molecular weight plant polyphenolics (tannins) as biological antioxidants. J Argic Food Chem.

[32] Sueishi Y, Hori M, Kita M, Kotake Y (2011). Nitric oxide (NO) scavenging capacity of natural antioxidants. Food Chem.

[33] Susan TGL, Jay ZL (1990). Evaluation of the role of xanthine oxidase in myocardial reperfusion injury. J Biol Chem.

[34] Andrew SG, Martin SO (2006). Obesity and the role of adipose tissue in inflammation and metabolism. Am J Clin Nutr.

[CR35] The pre-publication history for this paper can be accessed here:http://www.biomedcentral.com/1472-6882/14/342/prepub

